# Mantle Cell Lymphoma Mimicking Rectal Carcinoma

**DOI:** 10.1155/2014/621017

**Published:** 2014-04-17

**Authors:** Engin Kelkitli, Hilmi Atay, Levent Yıldız, Ahmet Bektaş, Mehmet Turgut

**Affiliations:** ^1^Department of Hematology, Erzurum Region Training and Research Hospital, 25770 Erzurum, Turkey; ^2^Department of Hematology, Van Training and Research Hospital, 65000 Van, Turkey; ^3^Department of Pathology, 19 Mayıs University Medical School, 55200 Samsun, Turkey; ^4^Department of Gastroenterology, 19 Mayıs University Medical School, 55200 Samsun, Turkey; ^5^Department of Hematology, 19 Mayıs University Medical School, 55200 Samsun, Turkey

## Abstract

Mantle cell lymphoma (MCL) is a mature B-cell non-Hodgkin lymphoma. After the (11;14) translocation was identified as its constant finding in 1992, MCL was recognized as a separate subgroup of non-Hodgkin lymphoma (NHL). In MCL, extranodal involvement may be observed in the bone marrow, the spleen, the liver, and the gastrointestinal system (GIS). Cases of MCL that present with a massive and solitary rectal mass are rare in the literature. In this case report, our aim was to present an MCL patient with a rarely observed solitary rectal involvement mimicking rectal carcinoma and to discuss treatment options for this patient.

## 1. Introduction

Mantle cell lymphoma (MCL) is a mature B-cell non-Hodgkin lymphoma [1]. MCL is observed in 3–10% of non-Hodgkin lymphomas (Table 1). It is more common among men, and the median age at presentation is 60–65 [2]. This type of lymphoma is associated with the chromosomal translocation t(11,14)(q13;q32), which is responsible for cyclin D1 overexpression [3]. In MCL, extranodal involvement may be observed in the bone marrow, the spleen, the liver, and the gastrointestinal system (GIS) [4]. In this case report, we present a case of MCL with rectal bleeding mimicking rectal carcinoma.

## 2. Case Report

**Figure 1:**
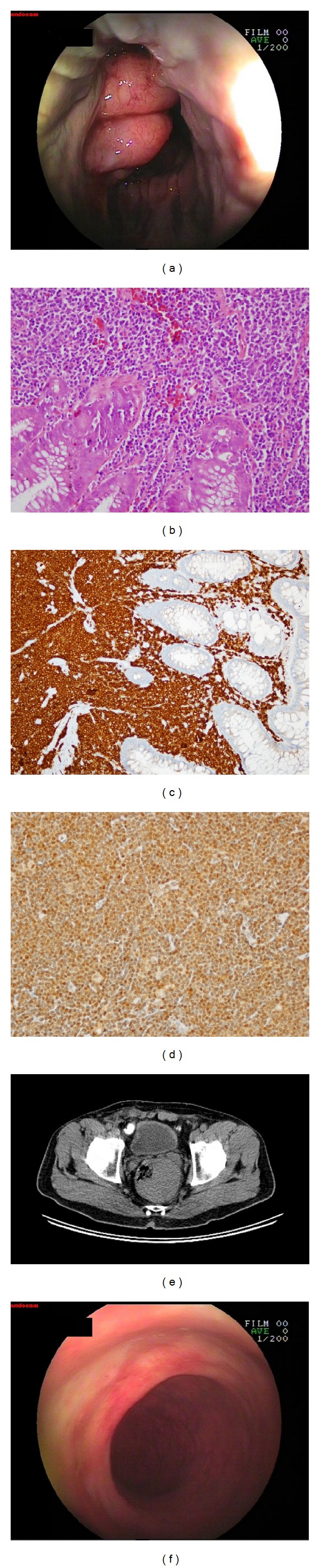
(a) Protruding mass adjacent to the anal canal. (b) Atypical lymphocytes infiltrating the hematoxylin-eosin-stained lamina propria and the surface epithelium in certain locations (original magnification ×40). (c) Cd79a-strained atypical lymphocytes infiltrating the lamina propria and the surface epithelium in certain locations (original magnification ×40). (d) Cyclin D1-positive atypical lymphocytes infiltrating the lamina propria (original magnification ×40). (e) Abdominal tomography. The 6 × 8 cm mass on the left-side wall of the rectum and the soft tissue mass inside the rectum. (f) Film showing the healed anal canal after treatment.

A 58-year-old male patient was admitted to the gastroenterology department with complaints of rectal bleeding for the past three months. B symptoms were not observed in the anamnesis. No significant findings were identified during physical examination. The following blood values were obtained for the patient: Hb: 14.7 g/dL, white blood cells: 12,700/*μ*L (3700–10100/*μ*L), neutrophils: 8500/*μ*L (2100–6100/*μ*L), lymphocytes: 3500/*μ*L (1300–3500/*μ*L), and platelets: 350,000/*μ*L (150.000–450.000/*μ*L). Peripheral blood smear was normal. During colonoscopy, a protruding mass was observed in the distal of the rectum (Figure 1(a)). Biopsy from this mass contained abnormal, diffuse, atypical, small-sized, slightly irregular neoplastic lymphocytes infiltrating the tissue. During immunohistochemical staining, CD3+, CD5+, CD20+, CD19+, CD43+, CD45+, Cd79a+, and BCL2+ and cyclin D1+, CD23−, CD10− staining were observed (Figures 1(b), 1(c), and 1(d)). No lymphadenopathies were observed in the thorax and neck CTs. Abdominal tomography revealed a 6 × 8 cm mass on the left-side wall of the rectum and a soft tissue mass inside the rectum (Figure 1(e)). Cytogenetic analysis was performed. The translocation t(11;14)(q13:q32) was detected. Based on these findings, the patient was diagnosed with MCL. The bone marrow biopsy showed a diffuse infiltrate composed of small, slightly irregular lymphocytes with condensed chromatin so that lymphoma involvement in the bone marrow was identified. Immunophenotypic characteristics of the cells are CD5+; CD20+; CD19+; and cyclin D1+, CD23−, and CD10−. Upper tract endoscopy was normal. According to the MCL international prognostic index (MIPI) score, the patient was considered as low risk. The patient was started on an R-CHOP (rituximab, cyclophosphamide, adriamycin, vincristine, and prednol) chemotherapy protocol. After significant regression was observed in the rectal mass during the evaluations performed following the 4th cycle, the chemotherapy protocol was completed and ended on the 8th cycle. Full response was observed in tomographies taken after the treatment and bone marrow infiltration regressed. In the control colonoscopy, the mass on the distal of the rectum had disappeared (Figure 1(f)). One year later, control tomographies of the patient identified a 6 × 8 cm mass surrounding the rectum. Biopsy was taken from a 3 cm protruding ulcerovegetative mass on the anal canal by rectoscopy. The biopsy results identified mantle cell lymphoma. At relapse peripheral blood smear, bone marrow biopsy, and upper tract endoscopy were normal. It was considered as local relapse. The patient was started on hyper-CVAD/MA (fractionated cyclophosphamide, vincristine, doxorubicin, and dexamethasone, alternating with high doses of methotrexate and cytarabine) plus rituximab and velcade chemotherapy protocol. The patient's treatment is currently ongoing.

## 3. Discussion

In 1961, Cornes reported multiple lymphomatous polyposis (MLP) involvement in the GIS for patients with lymphoma. In 1984, Isaacson et al. described the mantle cell form of the neoplastic lymphoid infiltration [5, 6]. In the study of Romaguera et al., upper GIS involvement was reported in 43% of MCL patients, while lower GIS involvement was reported in 88% of MCL patients. The most common sites of involvement are the gastric mucosa (74%) for the upper GIS and the colon and rectum (57.1% and 47.6%, resp.) for the lower GIS. Examination of lesions within the colon have shown that the MLP type is the most common form (80%), while protruding lesions are less common (18%) [7, 8]. Cases of MCL that present with a solitary rectal mass are rare in the literature. Romaguera et al. [6] showed that the great majority of MCL patients (88%) have lower GI involvement even in the absence of relevant clinical and endoscopic findings. However in this series of 71 patients with untreated MCL by Romaguera, there was no single patient presenting with a mass in the lower GI tract. In our case, the patient presented with a solitary mass in the rectum. Our case was diagnosed based on a rectal mass biopsy obtained for a prediagnosis of rectal cancer. One year after treatment, recurrence was observed in the rectum.

MCL demonstrates a clinically more aggressive course than other types of small B-cell lymphomas. Average survival period is 3–5 years [9, 10]. The effect of GIS involvement on the prognosis of MCL patients is currently a matter of debate. Within the context of a retrospective study, Iwamuro et al. investigated the effect of chemotherapy regimens on the median overall survival period of 35 MCL patients with GIS involvement and reported that, for patients below the ages of 60–65 and without any concomitant diseases, the hyper-CVAD/MA and PBSCT (peripheral blood stem cell transplantation) protocol was more effective than the other chemotherapy regimens [7]. Although R-CHOP chemotherapy was associated with high remission rates (75–96%) for these patients, the duration of remission was brief [11]. For patients with recurrence, new generation drugs are being used either singly or together with conventional chemotherapy protocols. One such drug is the proteasome inhibitor bortezomib. In the study of Romaguera et al., complete response was obtained in 17 out of 20 patients treated with a combination of bortezomib and R-hyper-CVAD/MA chemotherapy protocol; the toxicity of this combination was similar to that of other treatments. In another study Sachanas et al. showed that the rituximab plus chlorambucil (R-Chl) combination could be an effective therapeutic option as first-line treatment in MCL, especially for patients with indolent disease characteristics [12]. Leitch et al. showed that patients with limited-stage MCL had an improved progression-free survival (PFS) when treated with regimens including radiation therapy (RT), with a trend towards improved overall survival (OS). These results suggest a potentially important role for RT in limited-stage MCL [13]. As MCL is very uncommon in comparison to other lymphomas, multicenter prospective studies are necessary to develop new treatment regimens for patients with GIS involvement.

In conclusion, clinicians should consider during differential diagnosis that rectal masses observed in the colonoscopy of patients admitted for rectal bleeding could be associated with lymphoma involvement.

## Figures and Tables

**Table 1:** Mature B-cell neoplasms classification.

Mature B-cell neoplasms	%
Chronic lymphocytic leukemia/small lymphocytic lymphoma (CLL/SLL)	6.7
B-cell prolymphocytic leukemia (B-PLL)	1
Splenic marginal zone lymphoma	2
Hairy cell leukemia	2
Follicular lymphoma (FL)	20
Diffuse large B-cell lymphoma (DLBCL)	25–30
MALT lymphoma	7-8
Nodal marginal zone lymphoma	1.5–1.8
Mantle cell lymphoma	3–10
